# Threshold effect of plasma total homocysteine levels on cognitive function among hypertensive patients in China: A cross-sectional study

**DOI:** 10.3389/fneur.2022.890499

**Published:** 2022-08-18

**Authors:** Li Wang, Jianduan Chen, Junpei Li, Feng Hu, Yanyou Xie, Xinlei Zhou, Si Shen, Wei Zhou, Lingjuan Zhu, Tao Wang, Jianglong Tu, Huihui Bao, Xiaoshu Cheng

**Affiliations:** ^1^Department of Cardiovascular Medicine, The Second Affiliated Hospital of Nanchang University, Nanchang, China; ^2^Jiangxi Provincial Cardiovascular Disease Clinical Medical Research Center, Nanchang, China; ^3^Jiangxi Sub-center of National Clinical Research Center for Cardiovascular Diseases, Nanchang, China; ^4^Wuyuan Health Commission, Wuyuan, China; ^5^Center for Prevention and Treatment of Cardiovascular Diseases, The Second Affiliated Hospital of Nanchang University, Nanchang, China; ^6^Department of Neurology, The Second Affiliated Hospital of Nanchang University, Nanchang, China

**Keywords:** total homocysteine, cognitive function, Mini-Mental State Examination, threshold effect, hypertension

## Abstract

**Background:**

Increased plasma total homocysteine (tHcy) is an influencing factor of cognitive impairment in the general population. However, studies on the relationship between the risk of cognitive impairment and plasma tHcy levels in patients with hypertension are limited. This study aimed to explore the association between plasma tHcy levels and cognitive function assessed by MMSE scores among hypertensive patients in China.

**Methods:**

A total of 9,527 subjects from the Chinese Hypertension Registry Study participated in this study. Plasma tHcy levels were quantified by high-performance liquid chromatography using a fluorescence detector. Cognitive assessment was performed using the Mini-Mental State Examination (MMSE). Linear regression models, two piecewise linear regression models, and smoothing curve fitting were applied to determine the relationship between plasma tHcy levels and cognitive function.

**Results:**

This analysis included 9,527 Chinese hypertensive adults. Based on the results of linear regression models, a negative relationship was identified between plasma tHcy levels and MMSE scores [beta coefficient (β) per standard deviation (SD) increase: −0.26, 95% confidence interval (CI) −0.35, −0.16, *P* < 0.001]. The fully adjusted smooth curve fitting presented a nonlinear between plasma tHcy levels and MMSE scores. The threshold effect analysis showed that the inflection point of tHcy was about 27.1 μmol/L. The effect size [β (95% CI)] per SD increase in plasma tHcy concentrations on MMSE scores was −0.93 (−1.24, −0.6) on the left side and −0.07 (−0.24, 0.10) on the right side of the inflection point (*P*-value for log-likelihood ratio (LLR) test was <0.001). Moreover, subgroup analyses revealed that sex could influence the negative association between plasma tHcy levels and MMSE scores up to a specific threshold (*P*-value for interaction <0.001). Linear regression models indicated that there was an enhanced inverse association between tHcy levels and MMSE scores in female patients with tHcy concentrations less than 26.9 μmol/L compared to male patients with tHcy concentrations less than 32.0 μmol/L.

**Conclusions:**

Plasma tHcy levels had a threshold effect on MMSE scores among hypertensive patients in China. Increased plasma tHcy levels were independently inversely associated with cognitive decline among hypertensive patients with tHcy concentrations <27.1 μmol/L.

## Introduction

Based on world trends, the prevalence of dementia is estimated to increase dramatically by 2050 (1). Cognitive impairment, including mild cognitive impairment and dementia, can cause serious mental damage to patients and a huge financial burden on society. Given that there is no effective drug treatment for dementia, cognitive impairment is a huge economic burden on an aging society.

Cognitive impairment is a multifactorial disease with a variety of genetic and environmental etiologies, such as obesity, cerebral infarction, hypertension, and systemic illnesses (2). Most risk factors, including apolipoprotein E genotype, biological hormones, cardiovascular disease, and depression responsible for cognitive impairment, are evident in both sexes, but to date, many of these approaches have overlooked the role of sex difference when examining this association (3). Estrogens could chemically modulate homocysteine levels leading to a discrepancy in plasma total homocysteine (tHcy) levels between women and men (4, 5). The mechanisms are yet unclear, but the conjugation of estrogens could increase the formation of estrogen homocysteine conjugates, which results in lower levels of toxic homocysteine (4). Moreover, estrogens influence homocysteine metabolism including abnormities of anabolic and catabolic status, vitamin status, and the remethylation pathway (6).

Homocysteine is a sulfur-containing amino acid that is an important intermediate product in the metabolism of methionine and cysteine (7). Higher plasma tHcy concentrations have been frequently associated with cognitive impairment and dementia (8). Accumulating evidence suggests that hyperhomocysteinemia (HHcy) could deteriorate cognitive function among healthy older adults (9, 10). Compared to the healthy population, patients with Alzheimer's disease have increased plasma tHcy concentrations (11). Moreover, evidence from experimental studies has revealed that HHcy could cause platelet activation, endothelial dysfunction, oxidative stress, and chronic inflammation, contributing to the pathogenesis of dementia (12). Higher tHcy levels are associated with cerebral atrophy, a major pathological process in dementia (8).

In China, 245 million patients have hypertension, with a prevalence of 23.2% (13). Several research studies have suggested that elevated blood pressure, especially in elderly populations, is associated with cognitive impairment (14). However, no published studies to date have explored the effect of plasma tHcy concentrations on cognitive function among hypertensive patients. Considering that China bears a heavy burden of hypertension and that HHcy could worsen cognitive function, we investigated the association between plasma tHcy levels and cognitive function using the Mini-Mental State Examination (MMSE) test among hypertensive patients in China.

## Methods

### Study design and participants

The data analyzed in this study included the baseline of the ongoing China H-type Hypertension Registry in Wuyuan, Jiangxi Province of China from March 2018 (registration number: ChiCTR1800017274). Details regarding the design and methods are presented in previous publications (15, 16). This study recruited patients with hypertension aged >18 years. The exclusion criteria in this study were divided into the following aspects: (1) mental or neurological diseases leading to prevention of cooperation with the investigation; (2) planned to leave the area or could not be followed up later for some reasons; (3) subjects who deemed unsuitable to participate in the study by the physician. The protocol and data collection were approved by the Ethics Committee of the Institute of Biomedicine, Anhui Medical University (CH1059). All participants provided written informed consent prior to participating in the study. Out of 14268 subjects, 9527 agreed to participate in the study between March and August 2018. Hypertension was defined as systolic blood pressure (SBP) ≥140 mmHg and/or diastolic blood pressure (DBP) ≥90 mmHg, self-reported diagnosis of hypertension, or use of antihypertensive drugs (16). After excluding 34 patients without hypertension, 3945 subjects with incomplete MMSE questionnaires, and 762 subjects with a history of stroke, 9527 individuals completed the study (Supplement Figure 1).

### Data collection and indexes determination

At this baseline assessment, covariates were collected using a standardized questionnaire, such as sociodemographic characteristics including age, sex, education setting, smoking, and alcohol consumption; the other part was relevant medical history, including self-reported diabetes mellitus, medication usage, and cardiovascular diseases. Smokers who had smoked at least once a day for six months were classified as current cigarette smokers. Drinkers who drank at least once a week for one year were considered current drinkers.

Anthropometric measurements included body weight, height, SBP, and DBP. Body mass index (BMI) was calculated as body weight (kilograms) divided by height (meters squared). Blood pressure (BP) was measured four times on the subjects' right arm positioned at the level of the heart using electronic monitors (HBP-1300; Omron) after the participants rested for at least 5 minutes. SBP and DBP were calculated as the mean values of the last three readings. In our analysis, diagnosis of incident diabetes was defined as fasting blood glucose (FBG) greater than 7.0 mmol/L, and/or diagnosis of diabetes by doctors formerly.

The requirements for data collection were strict according to the standardization of the experimental process. Trained doctors and nurses collected all blood samples. Blood samples were collected utilizing venipuncture after an overnight fast of at least 12 h. Blood samples did not sit for more than 4 hours before analysis. They were kept on ice before analysis. They were not frozen and stored before analysis. Then they were obtained by centrifugation at 3,000 rpm for 10 min at 4°C. The levels of plasma total homocysteine (Hcy), fasting blood glucose (FBG), total cholesterol, triglyceride, high-density lipoprotein cholesterol (HDL-C), low-density lipoprotein cholesterol (LDL-C), and serum creatinine were measured using biochemical analysis instruments (Beckman Coulter) at the core laboratory of the National Clinical Research Center for Kidney Disease, Guangzhou, China. Moreover, the levels of plasma total homocysteine (Hcy) were measured using high-performance liquid chromatography with a fluorescence detector (17). The estimated glomerular filtration rate (eGFR) was assessed using the Chronic Kidney Disease Epidemiology Collaboration (CKD-EPI).

### Assessment of cognitive function

Currently, MMSE is the most commonly used cognitive screening tool and is widely used in the diagnosis of dementia (18). In this study, the Chinese version of MMSE, which measured cognitive function, has been widely used in the Chinese population due to its high reliability (19). The MMSE is a questionnaire with 30 questions concerning immediate memory, short-term memory, visuospatial memory, language, attention, calculation, and orientation. The MMSE is considered to have high interrater reliability and validity, and lower MMSE scores indicate poorer cognitive function.

### Statistical analysis

Data are presented as median (quartiles) for continuous variables and as count (percentage) for categorical variables. Baseline characteristics of study participants were described by quartiles of plasma tHcy levels. Comparisons among different plasma tHcy groups were performed using a nonparametric test (continuous variables) or chi-square test (categorical variables), accordingly.

To account for the potential nonlinearity in the association between plasma tHcy levels and MMSE scores, a generalized additive model (GAM) and smooth curve fitting (penalized spline method) were used. If nonlinearity was observed, we first used a recursive algorithm to calculate the inflection points and then constructed a two-segment binary linear regression model on both sides of the inflection points. Two piecewise linear regression models based on the beta coefficient (β) with their associated 95% confidence intervals (CI) were used to estimate the association between plasma tHcy levels and MMSE scores. The log-likelihood ratio (LLR) test was used to compare two piecewise logistic regression models to examine statistical significance.

Three different models were then used to examine the association between plasma tHcy levels and MMSE scores among hypertensive patients in China. The crude model was not adjusted for any confounder. Model I was adjusted for age, sex, and educational level. Model II was adjusted for age, sex, education, BMI, smoking status, alcohol consumption, diabetes, CHD, antihypertensive drugs, SBP, DBP, total cholesterol, triglycerides, HDL-C, LDL-C, and eGFR. We selected these confounders on the basis of their associations with the outcomes of interest when it was more than 10% (Supplementary Table 1). In addition, interaction and stratified analyses were used to evaluate whether covariates influenced the association between plasma tHcy levels and MMSE scores in different tHcy levels in a tabulated form.

All the analyses were performed using the statistical package R (http://www.R-project.org, The R Foundation) and the Empower (R; www.empowerstats.com; X&Y Solutions, Inc, Boston, MA, USA). All P-values were two-tailed, and *P* < 0.05 was considered statistically significant.

## Results

### Baseline characteristics of study participants

In total, 9,527 hypertensive participants (average age: 63.7 years, range 27–96 years; men, 48.0%) were included in the final data analysis. The baseline characteristics of the study population were described according to the quartiles of plasma tHcy levels (Table 1). Significant differences were found between quartiles of plasma tHcy levels in terms of age, the proportion of male and female patients, the proportion of current smoking and current drinking, education setting, levels of BMI and DBP, the prevalence of CHD and antihypertensive medication usage, the extent of total cholesterol, MMSE scores, triglycerides, HDL-C, LDL-C, and eGFR (all *P* < 0.05).

**Table 1:** Baseline characteristics of the study population according to quartiles of homocysteine.

**Characteristics**	**Total**	**Quartiles of homocysteine (**μ**mol/L)**				***P***
		** <12.4**	**12.4~14.8**	**14.8~18.9**	**≥18.9**	
Number of subjects (*n*)	9,527	2,380	2,374	2,391	2,382	
Male, *n* (%)	4,572 (48.0)	759 (31.9)	1,003 (42.2)	1,239 (51.8)	1,571 (66.0)	<0.001
Age, y	64.0 (57.0–70.0)	61.0 (54.0–66.0)	63.0 (56.0–69.0)	66.0 (59.0–71.0)	68.0 (61.0–74.0)	<0.001
BMI, kg/m^2^	23.5 (21.1–25.9)	23.9 (21.7–26.2)	23.6 (21.3–26.0)	23.4 (21.0–25.7)	23.0 (20.6–25.5)	<0.001
Current smoking, *n* (%)	2,500 (26.2)	423 (17.8)	558 (23.5)	701 (29.3)	818 (34.3)	<0.001
Current drinking, *n* (%)	2,132 (22.4)	453 (19.0)	507 (21.4)	594 (24.8)	578 (24.3)	<0.001
Education setting, *n* (%)						<0.001
Illiteracy	3,478 (36.5)	912 (38.3)	925 (39.0)	873 (36.5)	768 (32.2)	
Primary	4,024 (42.2)	935 (39.3)	948 (39.9)	1,011 (42.3)	1,130 (47.4)	
Secondary and above	2,025 (21.3)	533 (22.4)	501 (21.1)	507 (21.2)	484 (20.3)	
DBP, mmHg	89.3 (82.0–95.7)	90.3 (83.3–96.0)	89.7 (82.7–95.7)	88.7 (81.3–95.0)	88.3 (80.0–95.0)	<0.001
SBP, mmHg	146.0 (135.7–157.7)	145.7 (136.7–156.4)	145.7 (136.0–157.3)	146.3 (135.7–158.0)	146.3 (134.3–159.0)	0.626
Diabetes, *n* (%)	1,727 (18.1)	428 (18.0)	411 (17.3)	475 (19.9)	413 (17.3)	0.073
CHD, *n* (%)	533 (5.6)	104 (4.4)	128 (5.4)	146 (6.1)	155 (6.5)	0.008
Antihypertensive drugs, *n* (%)	5,798 (60.9)	1339 (56.3)	1387 (58.4)	1498 (62.7)	1574 (66.1)	<0.001
Triglyceride, mmol/L	1.5 (1.0–2.2)	1.5 (1.1–2.3)	1.5 (1.1–2.2)	1.5 (1.0–2.2)	1.4 (1.0–2.1)	<0.001
Total cholesterol, mmol/L	5.0 (4.4–5.8)	5.0 (4.3–5.7)	5.1 (4.4–5.8)	5.1 (4.4–5.9)	5.0 (4.3–5.8)	<0.001
HDL-C, mmol/L	1.5 (1.2–1.7)	1.4 (1.2–1.7)	1.5 (1.2–1.7)	1.5 (1.2–1.8)	1.4 (1.2–1.7)	<0.001
LDL-C, mmol/L	2.9 (2.4–3.4)	2.8 (2.4–3.4)	2.9 (2.4–3.4)	2.9 (2.4–3.4)	2.8 (2.4–3.4)	<0.001
eGFR, mL/(min·1.73m^2^)	91.0 (76.5–99.6)	98.6 (91.2–105.0)	93.8 (84.8–100.9)	87.2 (72.9–95.8)	76.3 (57.4–90.5)	<0.001
MMSE	23.0 (18.0–28.0)	24.0 (18.0–28.0)	24.0 (18.0–28.0)	23.0 (17.0–27.0)	23.0 (17.0–27.0)	0.029

Data presented as the median (quartiles) for continuous variables and percentage for categorical variables.

BMI, body mass index; SBP, systolic blood pressure; DBP, diastolic blood pressure; CHD, coronary heart disease; HDL-C, high-density lipoprotein cholesterol; LDL-C, low-density lipoprotein cholesterol; eGFR, estimated glomerular filtration rate; MMSE, Mini-mental State Examination.

### Threshold effect analysis of plasma tHcy levels on MMSE scores

**Figure 1:**
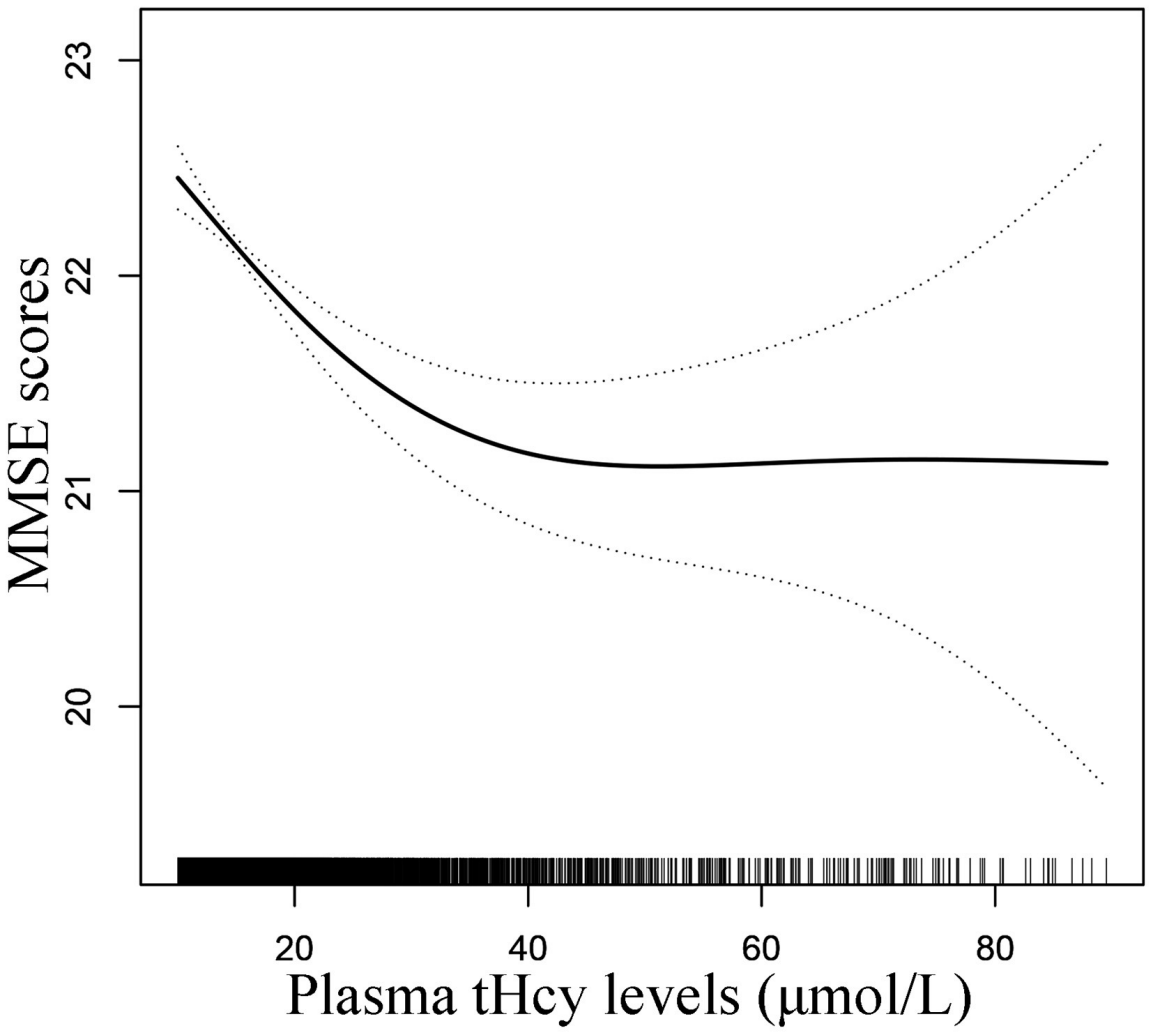
Smooth curve of correlation between plasma tHcy levels and MMSE scores. Smooth curve adjusted for age, sex, education, BMI, smoking status, alcohol consumption, diabetes, CHD, antihypertensive drugs, SBP, DBP, total cholesterol, triglycerides, HDL-C, LDL-C, and eGFR.

**Figure 2:**
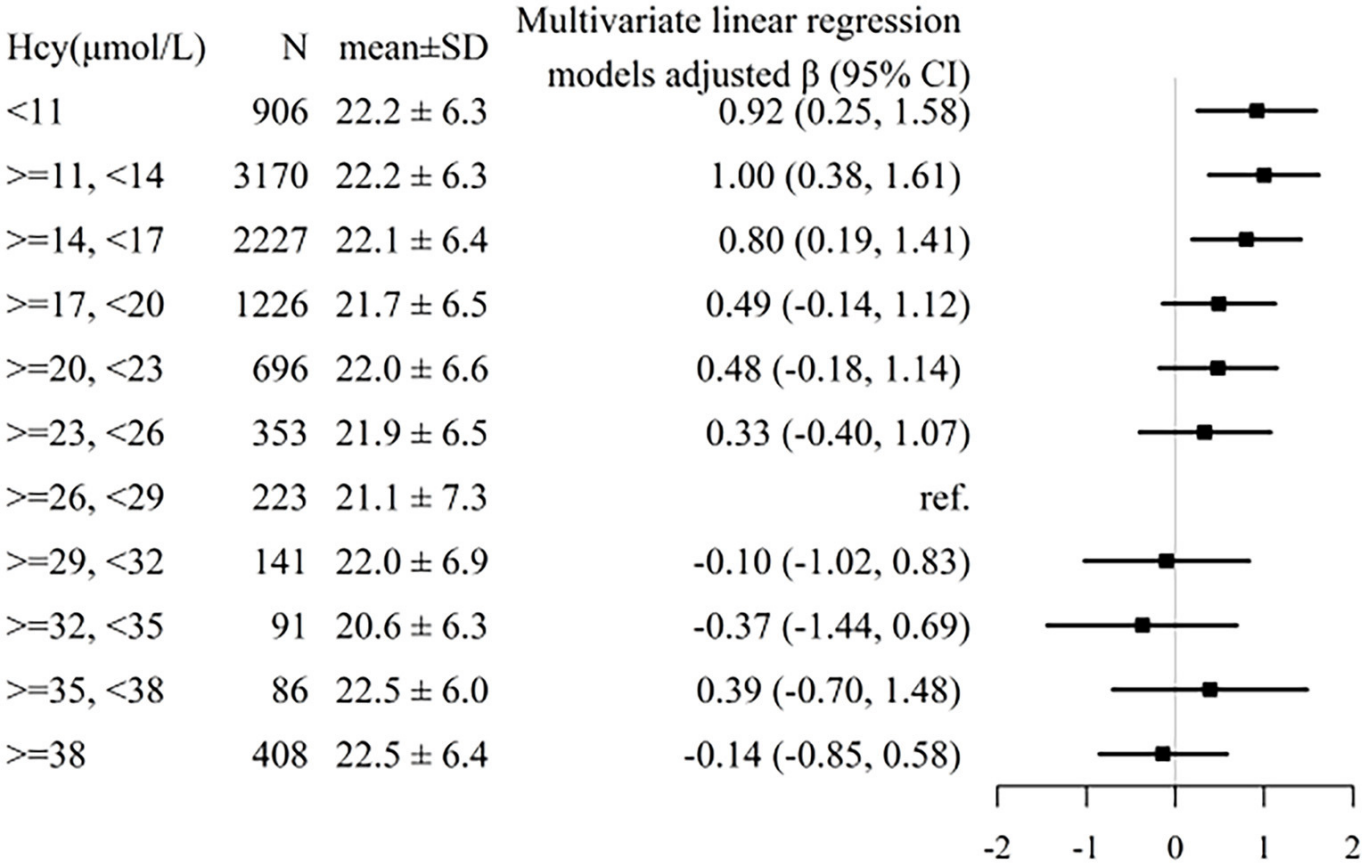
Association between plasma tHcy levels and MMSE scores stratified by 3 μmol/L intervals of tHcy concentrations. Individuals were divided into 3 μmol/L intervals of tHcy concentrations. Adjustment factors included age, sex, education, BMI, smoking status, alcohol consumption, diabetes, CHD, antihypertensive drugs, SBP, DBP, total cholesterol, triglycerides, HDL-C, LDL-C, and eGFR.

A generalized additive model and penalized spline method were used to assess the association between plasma tHcy levels and MMSE scores. Smooth curve fitting suggested there was a nonlinear association between plasma tHcy levels and MMSE scores (Figure 1). With the increase in plasma tHcy levels, MMSE scores first decreased and then leveled off. Visual inspection shows that the inflection point is ~27 μmol/L. Then, the two-piecewise binary logistic regression model was further fitted to the relationship between plasma tHcy levels and MMSE scores. The inflection point of plasma tHcy levels was 27.1 μmol/L (Supplementary Table 2). Effect size [β (95% CI)] per SD increase of plasma tHcy concentrations on MMSE scores was −0.93 (−1.24, −0.6) on the left side and −0.07 (−0.24, 0.10) on the right side of the inflection point (P-value for LLR test was <0.001, Table 2). Plasma tHcy levels were divided into 3 μmol/L intervals in a multivariable linear regression analysis model (Figure 2), which also indicated a threshold effect of plasma tHcy levels on MMSE scores.

**Table 2:** Threshold effect analysis of plasma tHcy levels on MMSE scores using piece-wise binary linear regression models.

**Plasma tHcy levels**	**Number of participants**	**MMSE scores**	**Crude model**		**Model I**		**Model II**	
			**β (95% CI)**	***P***	**β (95% CI)**	***P***	**β (95% CI)**	***P***
Per *SD* μmol/L increase	9527	22.1 ± 6.4	−0.01 (−0.14, 0.12)	0.913	−0.23 (−0.32, −0.14)	<0.001	−0.26 (−0.35, −0.16)	<0.001
**Inflection point**
<27.1 μmol/L	8670	22.1 ± 6.4	−0.54 (−0.93, −0.15)	0.006	−0.61 (−0.89, −0.33)	<0.001	−0.93 (−1.24, −0.6)	<0.001
≥27.1 μmol/L	857	22.0 ± 6.6	0.21 (−0.03, 0.45)	0.080	−0.08 (−0.25, 0.09)	0.346	−0.07 (−0.24, 0.10)	0.425
*P value* for LLR test			0.001		<0.001		<0.001	

tHcy, total homocysteine; MMSE, Mini-mental State Examination; β, beta coefficient; CI, confidence interval; SD, standard deviation; LLR, log-likelihood ratio.

Effect: MMSE scores; Cause: plasma tHcy levels per SD μmol/L increase. Model I was adjusted for age, sex, education. Model II was adjusted for age, sex, education, BMI, smoking status, alcohol consumption, diabetes, CHD, antihypertensive drugs, SBP, DBP, total cholesterol, triglycerides, HDL-C, LDL-C, and eGFR.

### Subgroup analyses by potential effect modifiers

**Figure 3:**
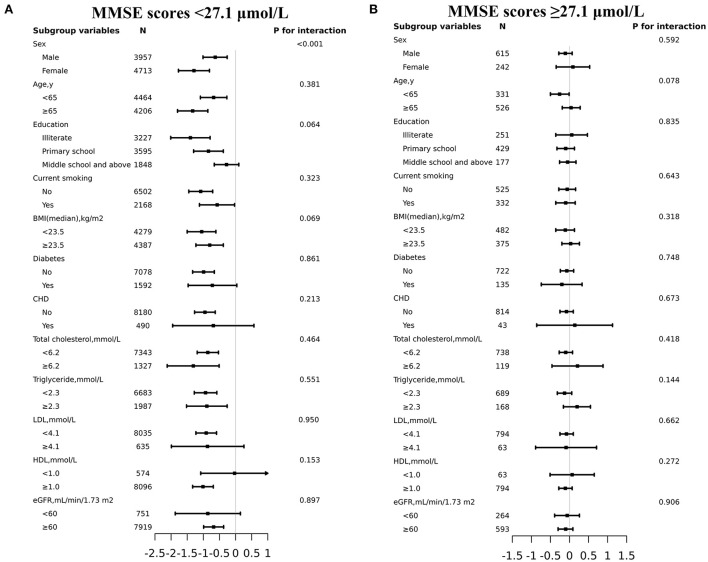
Effect size of plasma tHcy levels on MMSE scores in prespecified and exploratory subgroups. (**A**: plasma tHcy levels <27.1μmol/L, **B**: plasma tHcy levels≥27.1μmol/L). Each subgroup analysis adjusted for age, sex, education, BMI, smoking status, diabetes, CHD, antihypertensive drugs, SBP, DBP, total cholesterol, triglycerides, HDL-C, LDL-C and eGFR except for the stratifying variable.

To determine the effect of covariables in the relationship between plasma tHcy levels and MMSE scores, two groups of participants were stratified and interaction analyses were separated by the inflection point of tHcy (27.1μmol/L). There was an enhanced inverse relationship between tHcy levels and MMSE scores in male patients with tHcy concentrations less than 27.1 μmol/L (*P*-value for interaction <0.001, Figure 3). Linear regression models indicated that there was an enhanced inverse association between plasma tHcy levels and MMSE scores in female patients with tHcy concentrations <26.9 μmol/L compared to that in male patients with tHcy concentrations <32.0 μmol/L (*P*-value for interaction <0.001, Table 3). The effect size [β (95% CI)] per SD increase of tHcy concentrations on MMSE scores was −0.88 (−1.20, −0.55) on the left side and 0.05 (−0.25, 0.34) on the right of the inflection point in female subjects (*P*-value for LLR test was <0.001, Table 3). The effect size [β (95% CI)] per SD increase of tHcy concentrations on MMSE scores was −0.68 (−1.06, −0.31) on the left side and −0.07 (−0.32, 0.18) on the right of the inflection point in male subjects (*P*-value for LLR test was 0.003, Table 3).

**Table 3:** Threshold effect analysis of plasma tHcy levels on MMSE scores using piece-wise binary linear regression models by sex.

**Plasma tHcy levels**	**Number of participants**	**MMSE scores**	**Crude Model**		**Model I**		**Model II**	
			**β (95% CI)**	***P***	**β (95% CI)**	***P***	**β (95% CI)**	***P***
**Female**
Per *SD* μmol/L increase	4,955	19.4 ± 6.4	−0.74 (−0.91, −0.56)	<0.001	−0.28 (−0.41, −0.14)	<0.001	−0.35 (−0.49, −0.21)	<0.001
**Inflection point**
<26.9 μmol/L	4,707	19.5 ± 6.4	−1.96 (−2.35, −1.58)	<0.001	−0.54 (−0.84, −0.24)	<0.001	−0.88 (−1.20, −0.55)	<0.001
≥26.9 μmol/L	248	17.3 ± 6.8	0.14 (−0.25, 0.54)	0.472	−0.02 (−0.31, 0.26)	0.871	0.05 (−0.25, 0.34)	0.753
*P value* for LLR test			<0.001		0.001		<0.001	
**Male**
Per *SD* μmol/L increase	4,572	24.9 ± 5.0	−0.55 (−0.69, −0.40)	<0.001	−0.23 (−0.34, −0.11)	<0.001	−0.22 (−0.34, −0.10)	<0.001
**Inflection point**
<32.0 μmol/L	4,140	25.1 ± 4.9	−2.14 (−2.56, −1.72)	<0.001	−0.53 (−0.88, −0.18)	0.003	−0.68 (−1.06, −0.31)	<0.001
≥32.0 μmol/L	432	23.9 ± 5.4	−0.12 (−0.43, 0.20)	0.468	−0.06 (−0.31, 0.19)	0.629	−0.07 (−0.32, 0.18)	0.599
*P value* for LLR test			<0.001		0.001		0.003	

tHcy, total homocysteine; MMSE, Mini-mental State Examination; β, beta coefficient; CI, confidence interval; SD, standard deviation; LLR, log-likelihood ratio.

Effect: MMSE scores; Cause: plasma tHcy levels per SD μmol/L increase. Model I was adjusted for age, education. Model II was adjusted for age, education, BMI, smoking status, alcohol consumption, diabetes, CHD, antihypertensive drugs, SBP, DBP, total cholesterol, triglycerides, HDL-C, LDL-C, and eGFR.

## Discussion

This study showed a threshold effect of plasma tHcy levels on MMSE scores among hypertensive patients in China and the inflection point of tHcy was 27.1 μmol/L. There was an enhanced inverse relationship between tHcy levels and MMSE scores in female patients with tHcy concentrations of less than 26.9 μmol/L compared to male patients with tHcy concentrations <32.0 μmol/L.

A lot of evidence from experimental and clinical studies has revealed that elevated tHcy could cause cognitive decline, white matter damage, brain atrophy, neurofibrillary tangles, and dementia (8–12, 20–23). The Northern Manhattan study included 2871 stroke-free subjects and showed that elevated tHcy concentrations were independently associated with cognitive impairment (9). The odds of cognitive impairment were 2.8-fold higher in participants with HHcy than in those with plasma tHcy levels <10 μmol/L (20). In a cohort study consisting of healthy elderly people, cross-sectional analyses showed that increased tHcy levels were significantly associated with poorer performance on all neuropsychological tests and this result was further validated in a longitudinal study (21). Additionally, HHcy increased the risk of cognitive impairment and affects executive functioning, complex attention, cognitive flexibility, and memory in postmenopausal women (22). The study found modest inverse relationships between homocysteine levels and some indicators of cognitive function, including the global MMSE evaluation and the picture-association, verbal attention-span, and pattern-recognition tests (24).

Several possible mechanisms might contribute to the harmful effects of HHcy on cognitive decline. HHcy could increase S-adenosylhomocysteine (SAH) in the prefrontal cortex of Alzheimer's patients, which inhibits methyltransferases and is associated with cognitive impairment (25). Oxidative damage could result from interactions between highly reactive transition metals, such as copper, and oxidizing molecules, such as homocysteine in the brain (26). Increased copper or homocysteine levels in the elderly could promote significant oxidative damage to neurons and contribute to the development of Alzheimer's disease or related neurodegenerative conditions (26). HHcy and vascular contributed to cognitive impairment and dementia through myocyte proliferation, vessel wall fibrosis, impaired nitric oxide signaling, superoxide generation, and pro-coagulant actions (27).

To our knowledge, this is the first report on the threshold effect of plasma tHcy levels on MMSE scores in hypertensive populations, which might be attributed to geographic regional differences and higher levels of plasma tHcy in this crowd (28–30). A hypertensive population with higher tHcy levels was relatively common in China (28). The mean concentration of plasma tHcy was 17.9 μmol/L in our study, much higher than the mean tHcy was 9.8 μmol/L in a previous study (24).

According to the study results (Table 3), the inverse association of tHcy levels with MMSE was significant up to a specific threshold but disappeared above that in both sexes. However, the threshold is different according to sex [lower in females than males (26.9 vs. 32 μmol/L)], probably due to lower tHcy levels in females. In line with previous studies, homocysteine levels were significantly lower in females than in males patients (31). The differences may be related to estrogen, but since the average age of the study was in the post-menopausal range, any influence of estrogen would be diminished. Compared with female subjects, male patients had more risk factors such as smoking, drinking, and lower eGFR levels. Moreover, differences in B12, folate, and renal and possibly thyroid function may explain why the threshold was different according to sex. A limitation of this study was that major determinants of homocysteine levels were not controlled. These included B12 and folate status and thyroid function. Further research is needed to examine the relationship between sex, tHcy levels, and MMSE scores.

This study had several other limitations. First, the MMSE tests, as a screening test for the diagnosis of clinical cognitive impairment, are considered less sensitive and specific for mild cognitive impairment than other tests. Our study participants were more likely to be from a less educated rural population. Previous studies have shown that MMSE was a reliable tool for evaluating the cognitive function of people who are less educated (18, 19). Further studies combining MMSE and other tests, such as the Cognitive Capacity Screening Examination or Mini-Cog test, are needed to confirm this result. Second, this study was conducted on hypertensive Chinese patients; thus, we did not know whether this applies to other populations. Third, as with all observational studies, although we adjusted for multiple potential confounders, we cannot exclude the possibility of residual confounding factors including uncontrolled folate and vitamin B12 status. Finally, this study was a cross-sectional analysis and thus did not determine the causal relationship between plasma tHcy levels and cognitive decline. Intervention and prevention should be promoted to ameliorate cognitive symptoms in the elderly who are at risk of dementia, and more trials are required to investigate the preventive and therapeutic effects of homocysteine-lowering strategies on cognitive decline and to explore the underlying pathophysiological mechanisms.

## Conclusions

Our study suggests that there was a threshold effect of plasma tHcy levels on MMSE scores among hypertensive patients in China. Increased plasma tHcy levels were independently associated with a cognitive decline only among hypertensive patients with tHcy concentrations of <27.1 μmol/L. Moreover, this showed that the threshold differed according to sex, with female patients having a lower threshold than male patients. Further studies are needed to explore the underlying pathophysiological mechanisms on the threshold effect of plasma tHcy levels on the MMSE scores.

## Data availability statement

The original contributions presented in the study are included in the article/Supplementary material, further inquiries can be directed to the corresponding author/s.

## Ethics statement

The studies involving human participants were reviewed and approved by the Second Affiliated Hospital of Nanchang University. The patients/participants provided their written informed consent to participate in this study.

## Author contributions

LW and JC participated in literature search, data analysis, and data interpretation. LW wrote the manuscript. FH, JL, YX, XZ, SS, WZ, LZ, TW, and JT extracted and collected data, conceived the study, and participated in its design and coordination. HB and XC participated in the study design and provided critical revision. All authors read and approved the final manuscript.

## Funding

This work was supported by the Jiangxi Science and Technology Innovation Platform Project (20165BCD41005), the Jiangxi Provincial Natural Science Foundation (20212ACB206019), the Jiangxi Science and Technology Innovation Base Construction Project (20221ZDG02010), the Jiangxi Provincial Health Commission Science and Technology Project (202210495), and the Fund project of the Second Affiliated Hospital of Nanchang University (2016YNQN12034, 2019YNLZ12010, 2021efyA01, and 2021YNFY2024).

## Conflict of interest

The authors declare that the research was conducted in the absence of any commercial or financial relationships that could be construed as a potential conflict of interest.

## Publisher's note

All claims expressed in this article are solely those of the authors and do not necessarily represent those of their affiliated organizations, or those of the publisher, the editors and the reviewers. Any product that may be evaluated in this article, or claim that may be made by its manufacturer, is not guaranteed or endorsed by the publisher.

## Supplementary material

Supplementary Figure 1 Flow chart of this analysis. tHcy, total homocysteine; MMSE, Mini-mental State Examination.

Supplementary Table 1 Associations of covariates with MMSE scores. BMI, body mass index; SBP, systolic blood pressure; DBP, diastolic blood pressure; CHD, coronary heart disease; HDL-C, high-density lipoprotein cholesterol; LDL-C, low-density lipoprotein cholesterol; eGFR, estimated glomerular filtration rate; MMSE, Mini-mental State Examination; Ref, reference; β, beta coefficient; *CI*, confidence interval.

Supplementary Table 2 Saturation effect analysis of plasma tHcy levels and MMSE scores using Piece-wise logistic regression. Effect: tHcy, total homocysteine; Cause: MMSE, Mini-mental State Examination. Adjusted for age, sex, education, BMI, smoking status, alcohol consumption, diabetes, CHD, antihypertensive drugs, SBP, DBP, total cholesterol, triglycerides, HDL-C, LDL-C and eGFR. β, beta coefficient; CI, confidence interval; SD, standard deviation; LLR, log-likelihood ratio.
