# Supplementary figures and images for: Threshold effect of plasma total homocysteine levels on cognitive function among hypertensive patients in China: A cross-sectional study
Source: Front Neurol. 2022 Aug 18;13:890499. doi: 10.3389/fneur.2022.890499 (PMC9434013; doi:10.3389/fneur.2022.890499)

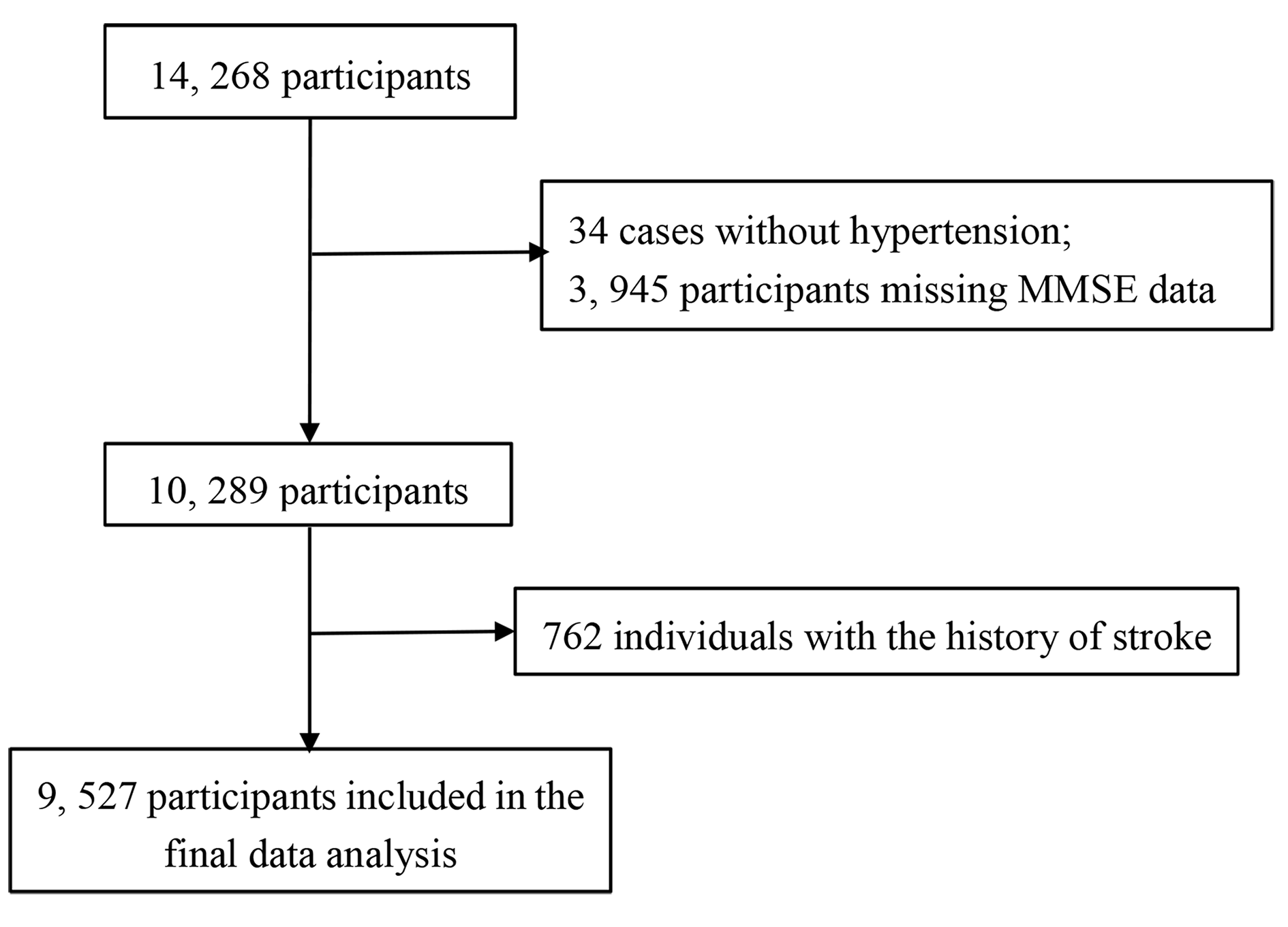

Supplement: Supplementary Figure 1 — Flow chart of this analysis. tHcy, total homocysteine; MMSE, Mini-mental State Examination. [file Image_1.TIF]
