# Supplementary material for: Threshold effect of plasma total homocysteine levels on cognitive function among hypertensive patients in China: A cross-sectional study
Source: Front Neurol. 2022 Aug 18;13:890499. doi: 10.3389/fneur.2022.890499 (PMC9434013; doi:10.3389/fneur.2022.890499)
Supplement: Supplementary Table 1 — Associations of covariates with MMSE scores. BMI, body mass index; SBP, systolic blood pressure; DBP, diastolic blood pressure; CHD, coronary heart disease; HDL-C, high-density lipoprotein cholesterol; LDL-C, low-density lipoprotein cholesterol; eGFR, estimated glomerular filtration rate; MMSE, Mini-mental State Examination; Ref, reference; β, beta coefficient; CI, confidence interval. [file Table_1.DOCX]

| **Covariates** | **MMSE scores** | |
| --- | --- | --- |
|  | ***β(95%CI)*** | ***P*-value** |
| Age (years) | -0.20 (-0.20, -0.20) | <0.001 |
| Sex, n(%) |  |  |
| Male | *Ref* |  |
| Female | -5.60(-5.80, -5.30) | <0.001 |
| SBP (mmHg) | -0.10 (-0.10, -0.10) | <0.001 |
| DBP (mmHg) | 0.10 (0.10, 0.10) | <0.001 |
| BMI (kg/m^2) | 0.20 (0.20, 0.30) | <0.001 |
| Current smoking, n(%) |  | 0.003 |
| No | *Ref* |  |
| Yes | 3.40 (3.10, 3.60) | <0.001 |
| Current Drinking, n(%) |  |  |
| No | *Ref* | 0.652 |
| Yes | 2.90 (2.70, 3.20) | <0.001 |
| Education |  |  |
| Illiterate | *Ref* |  |
| Primary school | 7.70 (7.50, 7.90) | <0.001 |
| Middle school and above | 11.2 (11.0, 11.5) | <0.001 |
| Diabetes |  |  |
| No | *Ref* |  |
| Yes | 0.00 (-0.30, 0.40) | 0.797 |
| CHD |  |  |
| No | *Ref* |  |
| Yes | -0.10 (-0.70, 0.40) | 0.628 |
| Antihypertensive drugs, n(%) |  |  |
| No | *Ref* |  |
| Yes | 0.50 (0.20, 0.80) | <0.001 |
| Total cholesterol (mmol/L) | -0.40 (-0.50, -0.20) | <0.001 |
| Triglyceride (mmol/L) | 0.30 (0.20, 0.40) | <0.001 |
| HDL-C (mmol/L) | -1.80 (-2.10, -1.40) | 0.192 |
| LDL-C (mmol/L) | -0.10 (-0.30, 0.10) | <0.001 |
| eGFR (ml/min/1.73m2) | 0.00 (0.00, 0.00) | <0.001 |

**Supplementary Table Legends**

| **Supplementary Table 1. Associations of covariates with** **MMSE scores.** |
| --- |

Abbreviations: BMI, body mass index; SBP, systolic blood pressure; DBP, diastolic blood pressure; CHD, coronary heart disease; HDL-C, high-density lipoprotein cholesterol; LDL-C, low-density lipoprotein cholesterol; eGFR, estimated glomerular filtration rate; MMSE, Mini-mental State Examination; Ref, reference; β, beta coefficient; CI, confidence interval.
