# Supplementary material for: Threshold effect of plasma total homocysteine levels on cognitive function among hypertensive patients in China: A cross-sectional study
Source: Front Neurol. 2022 Aug 18;13:890499. doi: 10.3389/fneur.2022.890499 (PMC9434013; doi:10.3389/fneur.2022.890499)
Supplement: Supplementary Table 2 — Saturation effect analysis of plasma tHcy levels and MMSE scores using Piece-wise logistic regression. Effect: tHcy, total homocysteine; Cause: MMSE, Mini-mental State Examination. Adjusted for age, sex, education, BMI, smoking status, alcohol consumption, diabetes, CHD, antihypertensive drugs, SBP, DBP, total cholesterol, triglycerides, HDL-C, LDL-C and eGFR. β, beta coefficient; CI, confidence interval; SD, standard deviation; LLR, log-likelihood ratio. [file Table_2.DOC]

| **Supplementary Table 2. Saturation effect analysis of** **plasma** **tHcy levels and MMSE scores using Piece-wise logistic regression.** | | | | | | |
| --- | --- | --- | --- | --- | --- | --- |
| Inflection point of Plasma tHcy | Number of participants | MMSE scores | Effect size (*β*) | *95%CI* | P-value | LLR test |
| <27.1 μmol/L | 8670 | 22.1 ± 6.4 | -0.93 | (-1.24, -0.6) | <0.001 | <0.001 |
| ≥27.1μmol/L | 857 | 22.0 ± 6.6 | -0.07 | (-0.24, 0.10) | 0.425 |
| Effect: tHcy, total homocysteine; Cause: MMSE, Mini-mental State Examination.  Adjusted for age, sex, education, BMI, smoking status, alcohol consumption, diabetes, CHD, antihypertensive drugs, SBP, DBP, total cholesterol, triglycerides, HDL-C, LDL-C and eGFR. Abbreviations: β, beta coefficient; CI, confidence interval; SD, standard deviation; LLR, log-likelihood ratio. | | | | | | |
|
